# Induction of Apoptosis by Isoalantolactone in Human Hepatocellular Carcinoma Hep3B Cells through Activation of the ROS-Dependent JNK Signaling Pathway

**DOI:** 10.3390/pharmaceutics13101627

**Published:** 2021-10-06

**Authors:** Min Yeong Kim, Hyesook Lee, Seon Yeong Ji, So Young Kim, Hyun Hwangbo, Shin-Hyung Park, Gi-Young Kim, Cheol Park, Sun-Hee Leem, Su Hyun Hong, Yung Hyun Choi

**Affiliations:** 1 Department of Biochemistry, Dong-eui University College of Korean Medicine, Busan 47227, Korea; ilytoo365@deu.ac.kr (M.Y.K.); 14769@deu.ac.kr (H.L.); 14602@deu.ac.kr (S.Y.J.); 14731@deu.ac.kr (S.Y.K.); hbhyun2003@naver.com (H.H.); 2 Anti-Aging Research Center, Dong-eui University, Busan 47340, Korea; 3 Department of Pathology, Dong-eui University College of Korean Medicine, Busan 47227, Korea; omdpark@deu.ac.kr; 4 Department of Marine Life Science, College of Ocean Sciences, Jeju National University, Jeju 63243, Korea; immunkim@jejunu.ac.kr; 5 Division of Basic Sciences, College of Liberal Studies, Dong-Eui University, Busan 47340, Korea; parkch@deu.ac.kr; 6 Department of Biomedical Sciences, College of Natural Sciences, Dong-A University, Busan 49315, Korea; shleem@dau.ac.kr; 7 Department of Health Sciences, The Graduated of Dong-A University, Busan 49315, Korea

**Keywords:** Isoalantolactone, HCC Hep3B cells, apoptosis, ROS, JNK

## Abstract

Isoalantolactone (IALT) is one of the isomeric sesquiterpene lactones isolated from the roots of *Inula helenium* L. IALT is known to possess various biological and pharmacological activities, but its anti-cancer mechanisms are not well understood. The aim of the present study was to investigate the anti-proliferative effects of IALT in human hepatocellular carcinoma (HCC) cells and to evaluate the potential anti-cancer mechanisms. Our results demonstrated that IALT treatment concentration-dependently suppressed the cell survival of HCC Hep3B cells, which was associated with the induction of apoptosis. IALT increased the expression of death-receptor-related proteins, activated caspases, and induced Bid truncation, subsequently leading to cleavage of poly (ADP-ribose) polymerase. In addition, IALT contributed to the cytosolic release of cytochrome c by destroying mitochondrial integrity, following an increase in the Bax/Bcl-2 expression ratio. However, IALT-mediated growth inhibition and apoptosis were significantly attenuated in the presence of a pan-caspase inhibitor, suggesting that IALT induced caspase-dependent apoptosis in Hep3B cells. Moreover, IALT activated the mitogen-activated protein kinases signaling pathway, and the anti-cancer effect of IALT was significantly diminished in the presence of a potent c-Jun N-terminal kinase (JNK) inhibitor. IALT also improved the generation of intracellular reactive oxygen species (ROS), whereas the ROS inhibitor significantly abrogated IALT-induced growth reduction, apoptosis, and JNK activation. Furthermore, ROS-dependent apoptosis was revealed as a mechanism involved in the anti-cancer activity of IALT in a 3D multicellular tumor spheroid model of Hep3B cells. Taken together, our findings indicate that IALT exhibited anti-cancer activity in HCC Hep3B cells by inducing ROS-dependent activation of the JNK signaling pathway.

## 1. Introduction

Hepatocellular carcinoma (HCC) is the most commonly diagnosed primary liver cancer that arises from hepatocytes. The incidence of HCC has increased markedly in developing countries over the past few decades, and the resulting mortality rate is much higher than in developed countries [1,2]. The development of HCC is associated with chronic liver damage, such as cirrhosis, and is a complex process involving inflammation, oxidative stress, cell damage and excessive proliferation, resulting in changes in several carcinogenic pathways [3,4]. Although infection with hepatitis B and C viruses is a major cause of the increased prevalence of HCC, consumption of contaminated food, cirrhosis related to heavy drinking, and nonalcoholic fatty liver disease also act as risk factors [3,5].

Clinically, these patients are usually treated with surgical resection, radiotherapy, and chemotherapy, but the recurrence rate of the tumor after treatment is still very high [3,5]. Sorafenib, a multiple-target tyrosine kinase inhibitor that exhibits apoptosis, mitigates angiogenesis, and suppresses tumor cell proliferation, has been the mainstay of the first pharmacological treatment for HCC in the past decade [6,7]. Sorafenib suppresses tumor cell proliferation by inhibiting Raf-1, B-Raf, and kinase activity in the Ras/Raf/MEK/ERK signaling pathways. In addition, sorafenib is capable of targeting platelet-derived growth factor receptor, vascular endothelial growth factor receptor 2, hepatocyte factor receptor, and other proteins to inhibit tumor angiogenesis [7,8]. Furthermore, regorafenib, cabozantinib, and ramucirumab have received approval as second-line treatments after sorafenib [9]. Besides these molecular-targeted therapies, checkpoint inhibitors nivolumab have also opened new strategies for the treatment of HCC [10]. Although several adjuvant chemotherapies used to reduce recurrence rates have improved survival in HCC patients, these drugs have serious complications and toxic side effects [11,12]. Therefore, it is urgent to understand the basic mechanism of HCC development and discover safe and effective therapeutic agents with fewer side effects that can inhibit the proliferation of HCC cells.

Apoptosis, a well-defined type of programmed cell death, is an essential mechanism for maintaining cellular homeostasis and tissue development in multicellular organisms. In particular, the escape of cancer cells from apoptosis is one of the hallmarks of cancer [13,14]. Therefore, increasing cancer cell apoptosis is recognized as a representative strategy for the treatment of all types of cancer, including HCC. Apoptosis can be broadly divided into death receptor (DR)-mediated extrinsic and mitochondria-mediated intrinsic pathways. These two pathways are critically regulated by a variety of intracellular apoptosis regulators and signaling pathways, which can be switched by inhibiting key steps in the processes [15,16]. Among these, the mitogen-activated protein kinases (MAPK) pathway is involved in multiple cellular processes, including proliferation, differentiation, and apoptosis [17,18,19]. On the other hand, while adequate levels of reactive oxygen species (ROS) play an important role as intracellular signaling molecules under normal conditions, their abnormal accumulation induces oxidative stress in cells and promotes apoptosis [20,21]. Moreover, many earlier studies demonstrated that the activation of the MAPK signaling pathway was associated with excessive ROS production [22,23,24]. These observations suggest that blocking MAPK signaling while promoting ROS production may be a potential approach for inducing apoptosis in cancer cells.

A source of broad pharmaceutical potential, Chinese herbal medicine is used to treat various diseases and is considered one of the most important sources of anti-cancer drugs. Sesquiterpene lactone compounds, a large and diverse group of chemicals containing a lactone ring, come from many medicinal plants and are reported to have diverse pharmacological actions [25,26]. Among them, isoalantolactone (IALT) was first purified from the roots of *Inula helenium* L. (the Elecampane family), which is widely used as a traditional prescription for the treatment of various diseases in East Asian countries, including China, Korea, and Japan [27,28,29]. Recent reports have shown that IALT has multiple pharmacological potentials, including antimicrobial, anti-inflammatory, antioxidant, neuroprotective, anti-adipogenic, anti-osteoporotic, anti-diabetic, and immunological adjuvant activities [30,31,32,33,34,35]. IALT has also been extensively reported to exert anti-cancer effects by inducing apoptosis, cell cycle arrest and autophagy in several types of cancer cells, which was confirmed to be selective for cancer cells [36,37,38,39]. Mechanistically, activation or inhibition of various cellular signaling pathways, including nuclear factor-κB, phosphatidylinositol 3-kinase/Akt, and MAPK, and increased ROS production were found to be involved in the induction of IALT-induced cancer cell apoptosis [40,41,42,43,44,45,46,47,48,49,50]. However, the anti-cancer effects of IALT on HCC cells have not been studied yet. Recently, three-dimensional (3D) multicellular tumor spheroid (MTS) models have gained much interest to overcome the limitations of in vitro two-dimensional (2D) monolayer culture of tumor cells. To date, 2D monolayer culture-based models have dominated the evaluation of pre-clinical cancer drug efficacy, but they do not adequately reproduce the natural structure of the tumor, cell-cell interactions, or the microenvironment of the tumor mass in vivo [51,52]. Therefore, the results obtained from 2D monolayer culture systems were sometimes not predictive of in vivo efficacy and did not match the results of in vivo assays [53,54]. However, the 3D MTS model has the potential to mimic the complex 3D organization of in vivo tumor tissue, similar to native tumor tissue [52,55]. Therefore, in this study, we investigated the effect and mechanism of action on IALT-induced apoptosis using 2D monolayer and 3D MTS models of HCC Hep3B cells.

## 2. Materials and Methods

### 2.1. Chemicals and Reagents

**Figure 1:**
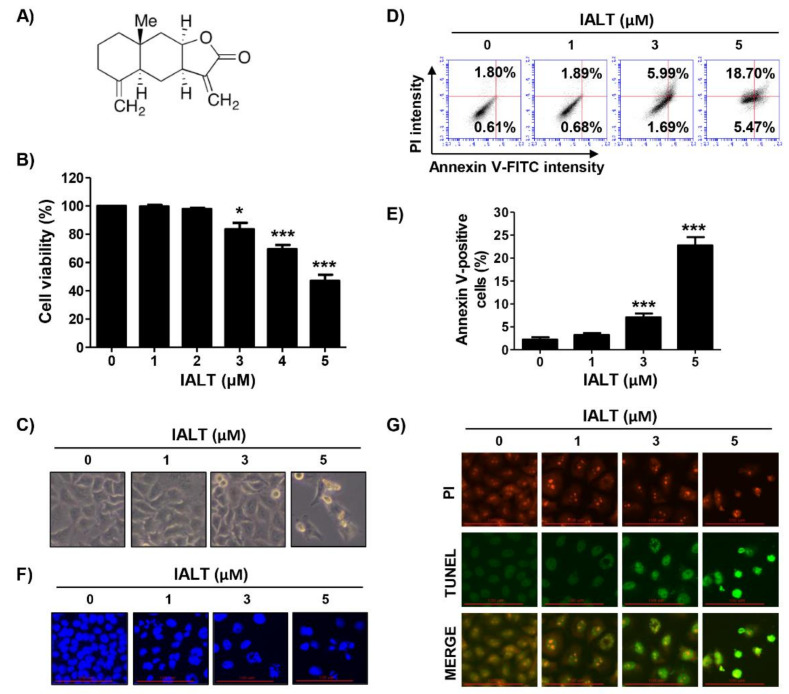
Inhibition of cell viability and induction of apoptosis by IALT in Hep3B cells. (**A**) Chemical structure of isoalantolactone (IALT). Cells were treated with the indicated concentrations of IALT for 48 h. (**B**) Cell viability was measured by the 3-(4,5-dimethylthiazol-2-yl)-2,5-diphenyltetrazolium bromide (MTT) assay. (**C**) Morphological changes were observed with a phase-contrast microscope. (**D**,**E**) Quantitative analysis of apoptosis induction by annexin V and propidium iodide (PI) staining was performed. (**D**) Representative profiles of flow cytometry results are presented. (**E**) The percentage of apoptotic cells was expressed as a percentage of the total number of annexin V-positive cells. (**F**) Nuclear morphological changes were observed under a fluorescence microscope using 4′,6′-diamidino-2-phenylindole (DAPI) staining (×400). (**G**) Representative images of terminal deoxynucleotidyl transferase dUTP nick end labeling (TUNEL) staining in control and IALT-treated cells are presented (×400). TUNEL-positive cells are shown as green fluorescence. PI staining is shown as red fluorescence. (**B**,**E**) Data are expressed as the mean ± SD (* *p <* 0.05 and *** *p <* 0.001 compared to untreated cells).

Dulbecco’s Modified Eagle’s Medium (DMEM), fetal bovine serum (FBS), and antibiotic mixtures were purchased from WelGENE Inc. (Gyeongsan, Korea). All consumables necessary for cell culture were also purchased from WelGENE Inc. IALT (Figure 1A), dimethyl sulfoxide (DMSO), 3-(4,5-dimethylthiazol-2-yl)-2,5-diphenyltetrazolium bromide (MTT), 4′,6′-diamidino-2-phenylindole (DAPI), N-acetyl-l-cysteine (NAC), N-benzyloxycarbonyl-Val-Ala-Asp-fluoromethylketone (z-VAD-fmk), SP600125, PD98059, and SB203580 were obtained from Sigma-Aldrich Chemical Co. (St. Louis, MO, USA). The fluorescein isothiocyanate (FITC)-conjugated annexin V and propidium iodide (PI) double staining kit and terminal deoxynucleotidyl transferase dUTP nick end labeling (TUNEL) apoptosis detection kit were obtained from Becton Dickinson (San Jose, CA, USA) and Promega Co. (Madison, WI, USA), respectively. 5,5′,6,6′-Tetrachloro-1,1′,3,3′-tetraethyl-imidacarbocyanine iodide (JC-1) and 2’,7’-dichlorofluorescein-diacetate (DCF-DA) were obtained from Invitrogen (Carlsbad, CA, USA). Caspase-3, -8, and -9 enzyme-linked immunosorbent assay (ELISA) kits were purchased from R&D Systems, Inc. (Minneapolis, MN, USA). Polyvinylidene difluoride (PVDF) membranes and mitochondrial fractionation kit were obtained from Schleicher & Schuell (Keene, NH, USA) and Active Motif, Inc. (Carlsbad, CA, USA), respectively. Primary antibodies were purchased from Cell Signaling Technology (Danvers, MA, USA), Santa Cruz Biotechnology, Inc. (Santa Cruz, CA, USA), and Abcam, Inc. (Cambridge, MA, USA). Horseradish peroxidase (HRP)-conjugated secondary antibodies and enhanced chemiluminescent (ECL) reagent were obtained from Santa Cruz Biotechnology, Inc. and Amersham Biosciences (Westborough, MA, USA), respectively. For 3D spheroid formation, Corning^®^ 96-well spheroid microplates purchased from Corning Inc. (Corning, NY, USA) were used. Tissue-Tek optimum cutting temperature (OCT) compound and spheroid dish were obtained from Sakura Finetek (Torrance, CA, USA) and SPL Life Science Co., Ltd. (Pocheon, Republic of Korea), respectively. All other reagents not specifically mentioned were obtained from Sigma-Aldrich Chemical Co.

### 2.2. Cell Culture and IALT Treatment

HCC Hep3B cells were obtained from the American Type Culture Collection (Manassas, VA, USA) and cultured in DMEM supplemented with 10% heat-inactivated FBS and antibiotics in a humidified incubator at 37 °C and 5% CO_2_. IALT was dissolved in DMSO to make a 20 mM stock solution and diluted to the desired final concentration in the medium at the time of use.

### 2.3. Cell Viability Assay

To measure cell viability, the MTT assay was performed, as described previously [56]. Briefly, the cells were incubated with different concentrations of IALT for 48 h or pre-treated with various pharmacological inhibitors (z-VAD-fmk, NAC, SP600125, PD98059 and SB203580) for 1 h and then treated with or without IALT. The treated cells were incubated in a medium containing 5 μg/mL MTT solution for 4 h. The medium was carefully discarded, and DMSO was added to each well and gently shaken for 10 min at room temperature (RT). The dissolved formazan was transferred to 96-well plates, and the absorbance was measured at 540 nm by an ELISA microplate reader (Beckman Coulter Inc., Brea, CA, USA) at the Core-Facility Center for Tissue Regeneration of Dong-eui University (Busan, Republic of Korea). In parallel, changes in cell morphology were observed with a phase-contrast microscope (Carl Zeiss, Oberkochen, Germany).

### 2.4. Detection of Apoptotic Morphological Changes

DAPI staining was used to investigate morphological changes in the nuclei to determine if apoptosis was induced. Briefly, the treated cells were harvested, washed with phosphate-buffered saline (PBS), and fixed for 10 min using 4% paraformaldehyde solution at RT, as previously described [57]. Subsequently, the cells were stained with DAPI solution (1 μg/mL) for 10 min in the dark and washed with PBS. Nuclear fluorescence was then photographed using a fluorescence microscope (EVOS FL Auto 2, Thermo Fisher Scientific, Inc., Agawam, MA, USA).

### 2.5. Determination of Apoptosis by Flow Cytometry

To analyze the degree of apoptosis, the collected cells were washed with PBS and subsequently stained with annexin V and PI solution for 20 min, according to the manufacturer’s protocol. The stained cells were detected using flow cytometry (Becton Dickinson, San Jose, CA, USA) and analyzed by Cell Quest Pro software version 5.2 (Beckman Coulter Inc., Brea, CA, USA). The apoptosis rate (%) was determined by dividing the number of annexin V-positive cells by the total number of observed cells, as previously described [58].

### 2.6. TUNEL Assay

For the TUNEL assay, the cells were fixed with 4% paraformaldehyde solution for 20 min at 4 °C, permeabilized using Triton X-100 (0.5% in PBS) for 10 min, and then washed twice with PBS. Subsequently, the TUNEL reaction mixture was applied to the fixed cells for 1 h at 37 °C, according to the manufacturer’s instructions. After washing with PBS three times, the cells were counterstained with propidium iodide (PI) solution for 15 min at RT in the dark and then washed with PBS to stain the nuclei. Finally, fluorescence-labeled damaged DNA strands (TUNEL-positive cells) were visualized under a fluorescence microscope.

### 2.7. Western Blot Analysis

The cells were lysed to extract whole proteins as previously described [59]. Mitochondrial and cytoplasmic proteins were isolated using a mitochondrial isolation kit, according to the manufacturer’s protocol. After denaturation, equal amounts of lysate were separated using sodium dodecyl sulfate-polyacrylamide gel electrophoresis and then blotted onto PVDF membranes. After blocking the membrane with 3% bovine serum albumin for 30 min at RT, membranes were incubated overnight at 4 °C with primary antibodies and then were incubated at RT for 2 h with HRP-conjugated secondary antibodies. Finally, the immunoreactive bands were detected using an ECL, according to the manufacturer’s procedure.

### 2.8. Caspase Activity Assay

Caspase activity ELISA assay kits were used to measure the proteolytic cleavage of fluorescent substrates to detect the activity of caspases. Briefly, after the cells were lysed using the provided lysis buffer, the supernatants were collected and reacted with the supplied reaction buffer containing a substrate for each caspase at 37 °C, according to the kit instructions. After reacting for 2 h, the optical density of the reaction mixtures of each sample was detected using an ELISA microplate reader and expressed as the relative fluorescence values of IALT-treated versus untreated samples.

### 2.9. Measurement of Mitochondrial Membrane Potential (MMP)

To measure MMP, JC-1 staining was performed. In brief, the collected cells were stained with 10 μM JC-1 for 30 min at 37 °C, following the manufacturer’s protocols. After washing the harvested cells with PBS, the MMP values were determined using a flow cytometer, as previously described [60].

### 2.10. Determination of ROS Generation

The generation of the total intracellular ROS produced in cells was measured using cell-permeable redox-sensitive dye DCF-DA, as previously described [61]. For this experiment, cells treated with IALT in the presence or absence of 20 mM NAC were reacted with 10 μM DCF-DA for 20 min at 37 °C in the dark, according to the manufacturer’s protocol. Immediately after the reaction, the levels of ROS production in each sample were analyzed using a flow cytometer.

### 2.11. Spheroid Formation Assay

Hep3B cells were grown at a density of 7 × 10^3^ cells/well in Corning^®^ spheroid microplates and incubated for 3 days at 37 °C in a humidified atmosphere of 5% CO_2_. To examine Hep3B spheroid formation, images of cultured spheroids were acquired 24 h later after seeding and every 24 h thereafter, and the spheroid size was calculated using Image J software (version 1.52a; NIH, Bethesda, MD, USA). For drug treatment, spheroids were pre-treated with or without 20 mM NAC for 1 h and then treated with the indicated concentrations of IALT for 6 days. The medium was replaced every 2~3 days.

### 2.12. Determination of ROS Levels in the Spheroids

To investigate the level of ROS generated in spheroids, 6 days after treatment, Hep3B spheroids were incubated in 10 μM DCF-DA for 20 min at 37 °C, washed with PBS, and then fixed with 3.7% paraformaldehyde in PBS for 10 min at RT. After being stained with DAPI solution (1 μg/mL) for 15 min at RT, spheroids were then washed twice with PBS, and intracellular DCF fluorescence was evaluated by a fluorescence microscope.

### 2.13. TUNEL Assay of the Spheroids

On day 6, spheroids were fixed with methanol for 10 min in −20 °C, washed in PBS, embedded in Tissue-Tek OCT compound, and then stored at −80 °C until used for experiments. Frozen sections were obtained at 7 μm thickness using a Cryostat (Leica CM1900, Leica, Wetzlar, Germany) in −25 °C to −30 °C. The sections were washed and permeabilized in 0.2% Triton X-100 in PBS for 5 min at RT. The sections were then washed and stained using the TUNEL apoptosis detection kit. The extent of the DNA breaks in apoptotic cells was evaluated by a fluorescence microscope.

### 2.14. Western Blot Analysis of the Spheroids

Hep3B (1 × 10^5^ cells per spheroid) cells were seeded into spheroid dishes. After 24 h of incubation, the medium was replaced to remove any cells that had landed outside the microwells. After seeding for 3 days, cells were pre-treated with 20 mM NAC for 1 h and then treated with 5 μM IALT for 6 days. The medium was replaced every 1~2 days. The spheroids were then collected and analyzed by Western blot analysis as described above.

### 2.15. Statistical Analysis

For statistical analysis, GraphPad Prism 5.03 statistical software (GraphPad Software, Inc., La Jolla, CA, USA) was used. All experimental values are expressed as mean ± standard deviation (SD). For statistical analysis, one-way analysis of variance and Tukey’s post-test were used to investigate differences between groups. If the *p*-value was less than 0.05, it was considered that a statistically significant difference was indicated.

## 3. Results

### 3.1. IALT Inhibits Cell Viability and Induces Apoptosis in Hep3B Cells

To investigate the effect of IALT on the proliferation of Hep3B cells, Hep3B cells were treated with various concentrations of IALT for 48 h, and then cell viability was measured by the MTT assay. As shown in Figure 1B, IALT significantly inhibited cell viability with increasing treatment concentrations, resulting in irregular cell contours, decreased cell density, and an increased number of isolated cells (Figure 1C). However, IALT does not affect cell growth in normal liver cells, including primary mouse hepatocytes and human Chang liver cells (Supplementary Figure S1A). Meanwhile, IALT also significantly suppressed cell viability in non-liver-derived carcinoma cells, including prostate cancer, breast cancer and lung cancer cell lines (Supplementary Figure S1B). These results suggest that IALT has more potential effect on the suppression of cell proliferation of various human carcinoma, including HCC, than normal cells.

Next, we examined whether the IALT-induced growth reduction of Hep3B cells was associated with apoptosis. The results of annexin V/PI double staining indicated that IALT concentration-dependently increased the frequency of apoptotic cells (Figure 1D,E). In addition, by performing DAPI and TUNEL staining, it was reconfirmed whether apoptosis was induced by IALT treatment. According to the results of fluorescence microscope observation, IALT treatment increased the number of condensed and fragmented nuclei and TUNEL-positive cells, which are characteristics of late-stage apoptosis, in a concentration-dependent manner (Figure 1F,G). Therefore, the results indicated that the inhibition of the proliferation of Hep3B cells by IALT was associated with the induction of apoptosis.

### 3.2. IALT Activates Caspases in Hep3B Cells

**Figure 2:**
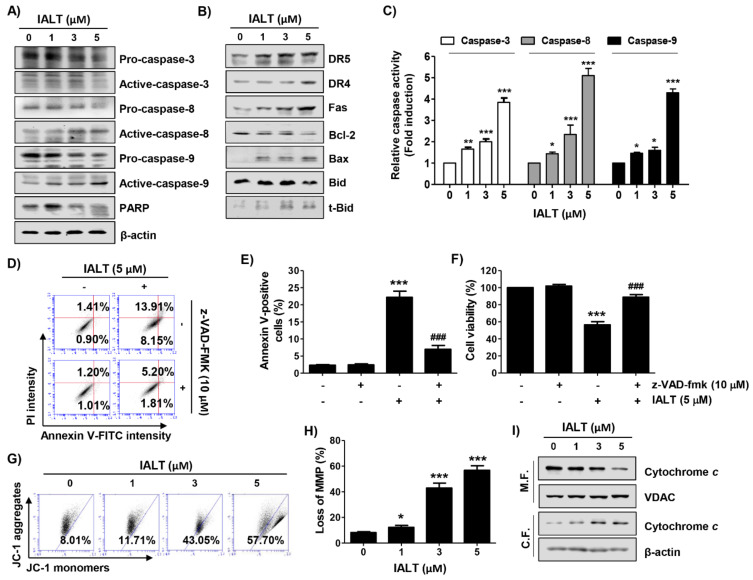
Activation of caspases, regulation of expression of Bcl-2 family proteins, loss of mitochondrial membrane potential (MMP), and cytosolic release of cytochrome *c* by IALT in Hep3B cells. Cells were treated with the indicated concentrations of IALT for 48 h (**A**–**C**, **G**–**I**) or treated with 10 μM N-benzyloxycarbonyl-Val-Ala-Asp-fluoromethylketone (z-VAD-fmk) for 1 h, and then treated with 5 μM IALT for another 48 h (**D**–**F**). (**A**,**B**) The results of Western blot analysis of the indicated proteins are presented. The immunoblot for β-actin served as a loading control. (**C**) Caspase activities in each sample were measured using colorimetric enzyme-linked immunosorbent assay (ELISA) assay kits. (**D**,**E**) Quantitative analysis of apoptosis induction by annexin V and PI staining was performed. (**D**) Representative profiles of flow cytometry results are presented. (**E**) The percentage of apoptotic cells was expressed as a percentage of the total number of annexin V-positive cells. (**F**) Cell viability was measured by the MTT assay. (**G**,**H**) Cells were stained with 5,5′,6,6′-Tetrachloro-1,1′,3,3′-tetraethyl-imidacarbocyanine iodide (JC-1) dye and then analyzed by flow cytometry for the extent of MMP loss. (**G**) Representative profiles of flow cytometry results. (**H**) The results of quantitative analysis of MMP loss are presented. (**I**) Mitochondrial and cytosolic proteins were isolated from cells, and cytochrome c expression was analyzed by Western blot analysis. Protein loading was confirmed by the analysis of voltage-dependent anion channel (VDAC) and β-actin expression in each protein extract. M.F., mitochondrial fraction; C.F., cytosolic fraction. (**C**,**E**,**F**,**H**) Data are expressed as the mean ± SD (* *p* < 0.05, ** *p* < 0.01 and *** *p* < 0.001 compared to untreated cells. ### *p* < 0.001 compared to 5 μM IALT-treated cells).

We next examined whether IALT activated the caspase signaling pathway and found that IALT increased the expression of the active forms of caspase-3, caspase-8, and caspase-9, and caused the degradation of poly (ADP-ribose) polymerase (PARP), a representative substrate protein degraded by the activated effector caspase (Figure 2A). Consistent with the immunoblotting results, the activities of the three caspases were also significantly increased by IALT (Figure 2C). Additionally, the expression of DR5, DR4, and Fas was concentration-dependently increased in response to IALT treatment (Figure 2B). Using the pan-caspase inhibitor, z-VAD-fmk, we further examined whether the IALT-induced apoptosis was caspase-dependent. As shown in Figure 2D–F, pretreatment with z-VAD-fmk significantly attenuated the induction of apoptosis and suppression of cell viability in IALT-treated Hep3B cells, but there was no significant difference following treatment with a necroptosis inhibitor (data not shown).

### 3.3. IALT Modulates the Expression of Bcl-2 Family Proteins and Increases Mitochondrial Dysfunction in Hep3B Cells

We subsequently assessed the changes in MMP and the expression of mitochondrial pathway-related proteins in IALT-treated Hep3B cells. As indicated in Figure 2B, IALT treatment increased the expression of pro-apoptotic Bax protein and the truncated form of BH3 interacting-domain death agonist (tBid), whereas the expression of anti-apoptotic Bcl-2 was decreased in a concentration-dependent manner. Furthermore, IALT triggered a concentration-dependent loss of MMP compared to untreated controls, and cytochrome c release from the mitochondria into the cytoplasm was enhanced in IALT-stimulated Hep3B cells (Figure 2G–I).

### 3.4. IALT Increases Intracellular ROS Generation in Hep3B Cells

**Figure 3:**
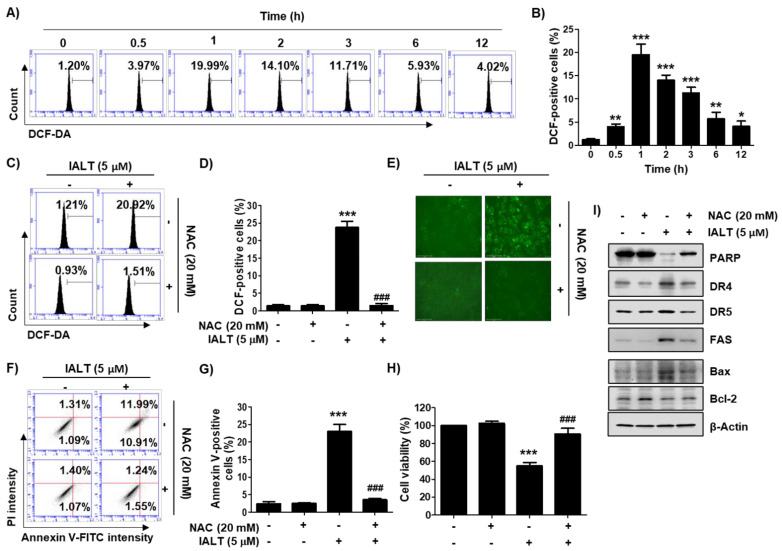
An increase in reactive oxygen species (ROS) production by IALT and its role on IALT-induced cytotoxicity in Hep3B cells. (**A**–**E**) Cells were treated with 5 μM IALT for the indicated times (**A**,**B**), or treated with or without 20 mM NAC for 1 h, and then treated with 5 μM IALT for 1 h (**C**–**E**). After staining with 2’,7’-dichlorofluorescein-diacetate (DCF-DA), ROS production was measured by flow cytometry analysis. (**A**,**C**) Representative profiles of flow cytometry results are presented. (**B**,**D**) The frequency of DCF-positive cells was expressed. (**E**) ROS generation was detected by a fluorescence microscope, and representative fluorescence micrographs depicting ROS generation are presented. (**F**–**I**) Cells were treated with 5 μM IALT for 48 h with or without 20 mM N-acetyl-L-cysteine (NAC) for 1 h. (**F**,**G**) Quantitative analysis of apoptosis induction by annexin V and PI staining was performed. (**F**) Representative profiles of flow cytometry results are presented. (**G**) The percentage of apoptotic cells was expressed as a percentage of the total number of annexin V-positive cells. (**H**) Cell viability was measured by the MTT assay. (**B**,**D**,**G**,**H**) Data are expressed as the mean ± SD (* *p* < 0.05, ** *p* < 0.01 and *** *p* < 0.001 compared to untreated cells. ^###^ *p* < 0.001 compared to 5 μM IALT-treated cells). (**I**) The results of Western blot analysis of the indicated proteins are presented. β-actin was used as the loading control.

DCF-DA staining was performed to evaluate the effect of IALT on intracellular ROS production, and the antagonistic effect was examined using the ROS scavenger, NAC. The flow cytometry analysis indicated that the ROS levels were markedly increased within 1 h of IALT treatment and gradually decreased over time (Figure 3A,B). However, IALT-induced ROS generation was significantly suppressed by pretreatment with NAC (Figure 3C,D), and the results of fluorescence microscopy were also consistent with these results (Figure 3E). To further evaluate whether IALT-induced cytotoxicity is associated with ROS production, we investigated the effect of NAC on IALT-induced apoptosis and growth inhibition and found that NAC pretreatment significantly protected cells against IALT-induced apoptosis (Figure 3F,G). Consistent with this, the IALT-induced reduction in cell viability was largely restored by blocking ROS production (Figure 3H). Furthermore, the expression of apoptosis-related proteins, which had been altered by IALT, was remarkably restored to the control level in the presence of NAC (Figure 3I).

### 3.5. IALT Activates the MAPK Signaling Pathway in Hep3B Cells

**Figure 4:**
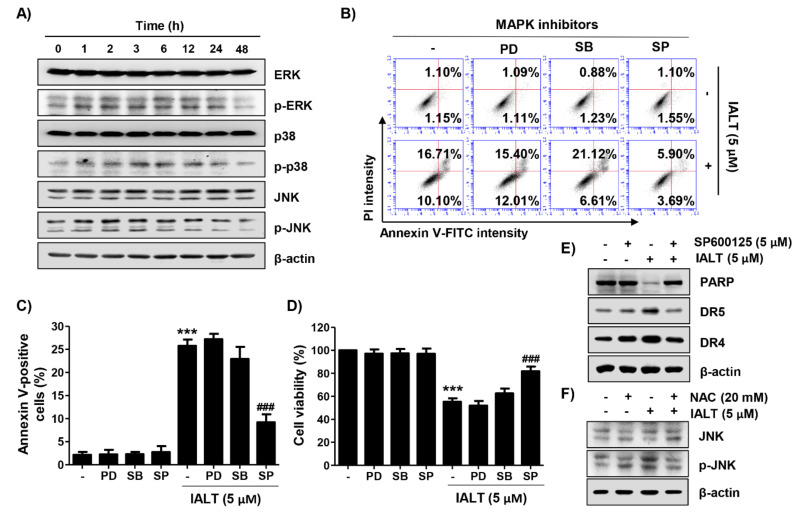
Activation of the mitogen-activated protein kinases (MAPK) signaling pathway by IALT in Hep3B cells. Cells were treated with 5 μM IALT for the indicated times (**A**), or pre-treated with MAPK inhibitors [25 μM PD98059 (PD), 2.5 μM SB203580 (SB) or 5 μM SP600125 (SP)] or 20 mM NAC for 1 h, and then treated with 5 μM IALT for another 48 h (**B–F**). (**A**) The expression of extracellular-signal-regulated kinase (ERK), p38, and c-Jun N-terminal kinase (JNK) proteins and their phosphorylated forms were evaluated by Western blot analysis. The immunoblot for β-actin served as a loading control. (**B**,**C**) Quantitative analysis of apoptosis induction by annexin V and PI staining was performed. (**B**) Representative profiles of flow cytometry results are presented. (**C**) The percentage of apoptotic cells was expressed as a percentage of the total number of annexin V-positive cells. (**D**) Cell viability was measured by the MTT assay. (**E**,**F**) The results of Western blot analysis of the indicated proteins are presented. The immunoblot for β-actin served as a loading control. (**C**,**D**) Data are expressed as the mean ± SD (*** *p* < 0.001 compared to untreated cells. ^###^ *p* < 0.001 compared to 5 μM IALT-treated cells).

Next, the influence of the MAPK signaling pathway contributing to the anti-cancer effect of IALT was evaluated. The results of the Western blot analysis illuminated that the levels of phosphorylated extracellular-signal-regulated kinase (p-ERK) and c-Jun N-terminal kinase (p-JNK) were increased within 1 h after treatment with 5 μM IALT without any change in their total proteins. On the other hand, the highest activation of p38 was observed at 3 to 4 h after IALT treatment and then gradually decreased, indicating that the MAPK signaling pathway was activated by IALT treatment (Figure 4A). Therefore, to explore whether IALT-mediated apoptosis in Hep3B cells was through the activation of the MAPK signaling pathway, we pre-treated with MAPK elements inhibitors for 1 h prior to exposure to IALT. As shown in Figure 4B,C, only the JNK inhibitor (SP600125), but not the ERK inhibitor (PD98059) and p38 inhibitor (SB203580), weakened IALT-induced apoptosis and inhibition of cell viability, demonstrating that IALT induced apoptosis in Hep3B cells through activation of JNK. Additionally, the result of the immunoblot assay showed that IALT-induced expression of DRs, as well as the degradation of PARP, were blocked when Hep3B cells were pre-treated with SP600125 (Figure 4E). Furthermore, IALT-induced JNK activation was dramatically diminished by co-treatment with NAC compared to treatment with IALT alone (Figure 4F), indicating that ROS acted as an upstream initiator inducing activation of JNK in IALT-treated Hep3B cells.

### 3.6. IALT Suppresses the Growth of Hep3B MTSs

**Figure 5:**
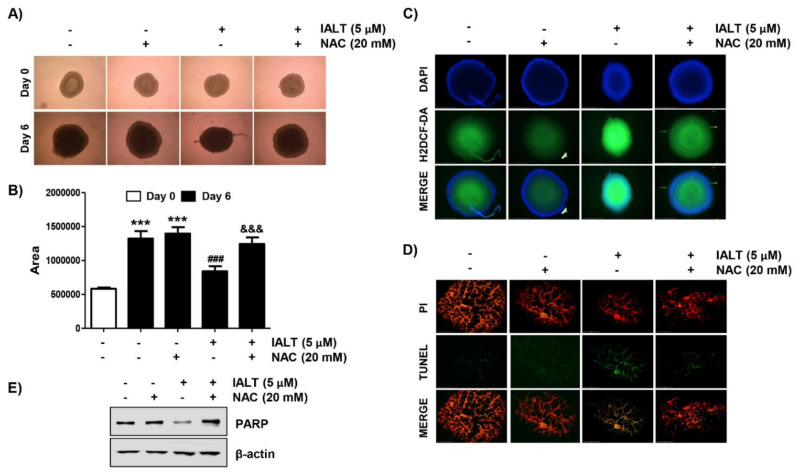
Effects of IALT on Hep3B cell growth in 3D spheroid model. Spheroids were formed for 3 days and treated with 5 μM IALT for 6 days or pre-treated with or without 20 mM NAC for 1 h and then treated with 5 μM IALT for further 6 days. (**A**) The images of representative spheroids obtained under a phase-contrast microscope on the final day of treatment (9 days after cell seeding) are presented. Images were taken at 200× magnification. (**B**) The spheroid size was calculated using Image J software. Data are expressed as the mean ± SD (*** *p* < 0.001 compared to day 0 spheroids. ^###^ *p* < 0.001 compared to untreated spheroids. ^&&&^ *p* < 0.001 compared to 5 μM IALT-treated spheroids). (**C**) Spheroids were stained with DCF-DA, and the generation of ROS was observed under a fluorescence microscope, and representative fluorescence micrographs depicting ROS generation are presented. (**D**) The sections of spheroids were stained using the TUNEL apoptosis detection kit and then observed under a fluorescence microscope. Images were taken at 400× magnification. (**E**) The levels of PARP were analyzed by Western blot analysis. β-actin was used as the loading control.

To confirm the anti-cancer activity of IALT in a more realistic in vitro model, we generated a 3D MTS model derived from Hep3B cells, and the effect of IALT on the growth of spheroids was investigated. To this end, IALT-treated spheroids were cultured for 6 days and monitored under an optical microscope. As shown in Figure 5A,B, IALT treatment reduced Hep3B spheroid growth, as illustrated by the decreased volume of tumor spheroids. Moreover, consistent with the results of 2D monolayer cultures, IALT exposure has been shown to increase the generation of ROS and the number of TUNEL positive cells in Hep3B spheroids, as evidenced by increased fluorescence intensity (Figure 5C,D), and the expression of PARP protein was decreased in IALT-treated spheroids (Figure 5E). However, these phenomena were markedly abolished in the presence of NAC. Taken together, these results imply that IALT can inhibit Hep3B spheroid growth ex vivo through the induction of apoptosis associated with ROS production, as in 2D monolayer cultures.

## 4. Discussion

Of the two main apoptosis pathways, the extrinsic pathway is mediated by the activation of caspase-8 according to the binding of death ligands and DRs [15,16]. The initiation of the intrinsic pathway is associated with mitochondrial dysfunction due to changes in the expression of Bcl-2 family proteins, which in turn leads to the cytosolic release of apoptogenic factors, such as cytochrome c to activate caspase-9 [62,63]. Therefore, caspase-8 and caspase -9 act as initiator caspases of the two apoptosis pathways, respectively, and activate executor caspases, including caspase-3 and caspase-7, completing apoptosis by the cleavage or degradation of various cellular substrates such as PARP [64,65]. As is well known, activation of caspase-8 can additionally cleave and convert Bid, a protein belonging to the Bcl-2 family, to tBid. tBid, in turn, translocates to the mitochondria, promoting permeability of the outer mitochondrial membrane [15,65]. In this study, we demonstrated that IALT activated both caspase-8 and caspase-9 and promoted mitochondrial dysfunction in HCC Hep3B cells. These results were accompanied by increased expression of DR-related regulators, upregulation of the Bax/Bcl-2 expression ratio, promotion of the cytosolic release of cytochrome c, Bid truncation, and the loss of MMP. In cells treated with IALT, the activity of caspase-3 was also greatly increased, and PARP degradation was observed. However, IALT-induced cytotoxicity was markedly suppressed by pretreatment with a pan-caspase inhibitor. Therefore, the current results demonstrate that IALT induced apoptosis by simultaneously activating the caspase-dependent extrinsic and intrinsic pathways through tBid-mediated crosstalk in Hep3B cells.

The MAPK signaling pathway is involved in a variety of cellular responses. In general, among MAPKs, ERK is known as a positive regulator that controls cell proliferation and differentiation, whereas the activation of JNK and p38 participates in the induction of apoptosis and responses to various cellular stresses [17,18,19]. However, the role of p38 and JNK in regulating cancer cell proliferation remains controversial. It is well known that ROS play critical roles as secondary messengers in several redox-sensitive intracellular signaling pathways. In particular, as an intrinsic apoptosis inducer, ROS act as major regulators of apoptosis by regulating the expression of Bcl-2 family proteins and the activity of MAPK family members and their downstream transcription factors [22,23]. One previous study reported that activation of p38 was an important determinant of IALT-induced apoptosis in pancreatic carcinoma cells [50]. Although the roles of ERK and JNK were not investigated in this study, the activation of p38 by IALT was ROS-dependent. Moreover, a recent study showed that IALT increased phosphorylation of p38 and JNK in breast cancer cells, but not in normal breast cells, and IALT-mediated apoptosis could be abolished by inhibitors of p38 and JNK [45]. In addition, several previous studies have shown that an increase in ROS production was involved in IALT-induced apoptosis in various types of cancer cells [40,42,43,48,49]. Therefore, we assessed whether the IALT-induced apoptosis of Hep3B cells was related to ROS and investigated the role of ROS in the activation of the MAPK signaling pathway. In the present study, ROS levels were markedly increased during the early stage of IALT treatment. We also found that IALT upregulated the levels of p-ERK, p-38 and p-JNK, meaning that the MAPK signaling pathway was activated by IALT treatment. Furthermore, SP600125, a specific inhibitor of JNK, ameliorated IALT-induced cytotoxic effects, whereas ERK and p38 inhibitors did not, suggesting that among MAPKs, the JNK signaling pathway mediates the apoptotic action of IALT in Hep3B cells. In addition, quenching ROS production significantly ameliorated IALT-induced apoptosis and viability reduction and blocked IALT-induced phosphorylation of JNK. These results revealed that IALT induced apoptosis in Hep3B cells by causing ROS-mediated oxidative damage and activation of the JNK signaling pathway.

As IALT induced apoptosis in Hep3B cells used in a traditional 2D cell culture model, we further investigated the effects of IALT in 3D spheroids derived from Hep3B cells to mimic the in vivo environment. According to our results, IALT significantly inhibited the growth of Hep3B spheroids, which was accompanied by an increase in DCF fluorescence intensity inside the spheroids. However, IALT-induced growth inhibition of spheroids, reduction of PARP expression, and induction of apoptosis were restored to control levels by NAC pretreatment. Although further studies are needed to elucidate the possible crosstalk between the ROS-mediated apoptosis pathway and the intracellular signaling pathway in 3D Hep3B MTSs, our results support the results of 2D cultures in which ROS served as upstream regulators in IALT-induced Hep3B cell apoptosis well.

## 5. Conclusions

**Figure 6:**
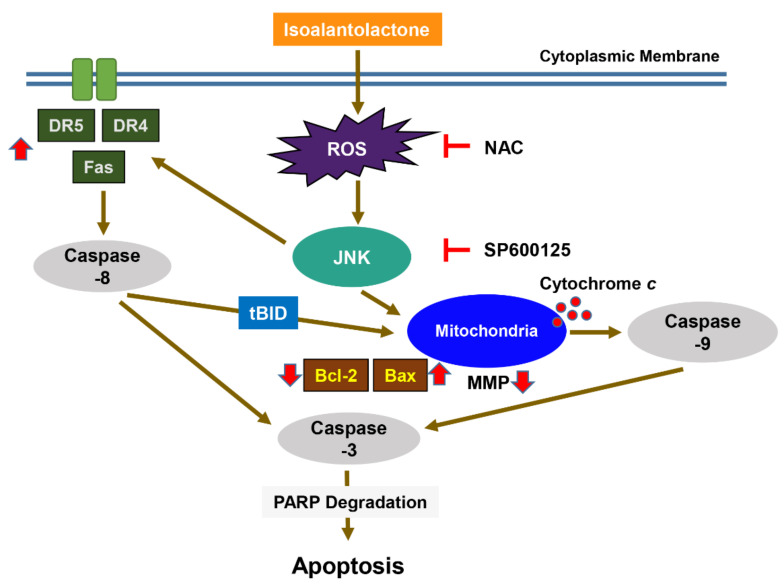
Graphical diagram denoting the mechanism of IALT-induced apoptosis in human HCC Hep3B cells. Exposure to IALT induces ROS-mediated activation of JNK and promotes the activation of extrinsic and intrinsic apoptosis pathways. Therefore, ROS act as upstream regulators of the JNK signaling pathway for apoptosis induction.

In summary, the present results suggest that ROS production by IALT played a key role in the induction of apoptosis in HCC Hep3B cells and acted as an upstream signal to activate the JNK signaling pathway Figure 6. IALT also effectively suppressed 3D Hep3B spheroid growth, suggesting that IALT may be a therapeutic candidate for the management of HCC. Although we identified that the partial role of the ROS/JNK pathway in IALT-induced HCC Hep3B apoptosis, we did not verify whether the ROS/JNK signaling pathway be correlated with other physiological processes. Therefore, further studies are required to determine whether the ROS/JNK signaling pathway is correlated with other physiological processes and the role of other cellular signaling pathways that may be involved in the anti-cancer activity of IALT. In addition, establishing the role of other intracellular organelles in cells involved in the ROS generation by IALT should also be a priority, and validation of the anti-cancer efficacy of IALT through in vivo animal experiments is needed.

## Data Availability

The datasets during and/or analyzed during the current study are available from the corresponding author on reasonable request.

## Supplementary Materials

The following are available online at https://www.mdpi.com/article/10.3390/pharmaceutics13101627/s1, Figure S1: The effect of IALT on cell viability in non-carcinoma cells and various types of carcinoma cells. (A) Non-carcinoma hepatocytes, including primary mouse hepatocytes and Chang liver cells, were treated with the indicated concentration of IALT for 48 h. (B) Three prostate carcinoma cell lines, including PC3, DU145, and LNCaP, and two breast cancer cell lines, MDA-MD-231 and MCF-7, and human lung adenocarcinoma A549 cells were treated with IALT. (A and B) Cell viability was measured by MTT assay. The data are expressed as mean ± standard deviation (SD) of three independent experiments. * *p* < 0.05, ** *p* < 0.01 and *** *p* < 0.001 vs. untreated control group.
