# Supplementary material for: Induction of Apoptosis by Isoalantolactone in Human Hepatocellular Carcinoma Hep3B Cells through Activation of the ROS-Dependent JNK Signaling Pathway
Source: Pharmaceutics. 2021 Oct 6;13(10):1627. doi: 10.3390/pharmaceutics13101627 (PMC8540929; doi:10.3390/pharmaceutics13101627)
Supplement: Supplementary file 1 [file pharmaceutics-13-01627-s001.zip › pharmaceutics-1405050-SM.pdf]

pharmaceutics

MDPI

Supplementary Material: Induction of Apoptosis by Isoalantolactone in Human Hepatocellular Carcinoma Hep3B Cells through Activation of the ROS-Dependent JNK Signaling Pathway

Min Yeong Kim, Hyesook Lee, Seon Yeong Ji, So Young Kim, Hyun Hwangbo, Shin-Hyung Park, Gi-Young Kim, Cheol Park, Sun-Hee Leem, Su Hyun Hong and Yung Hyun Choi

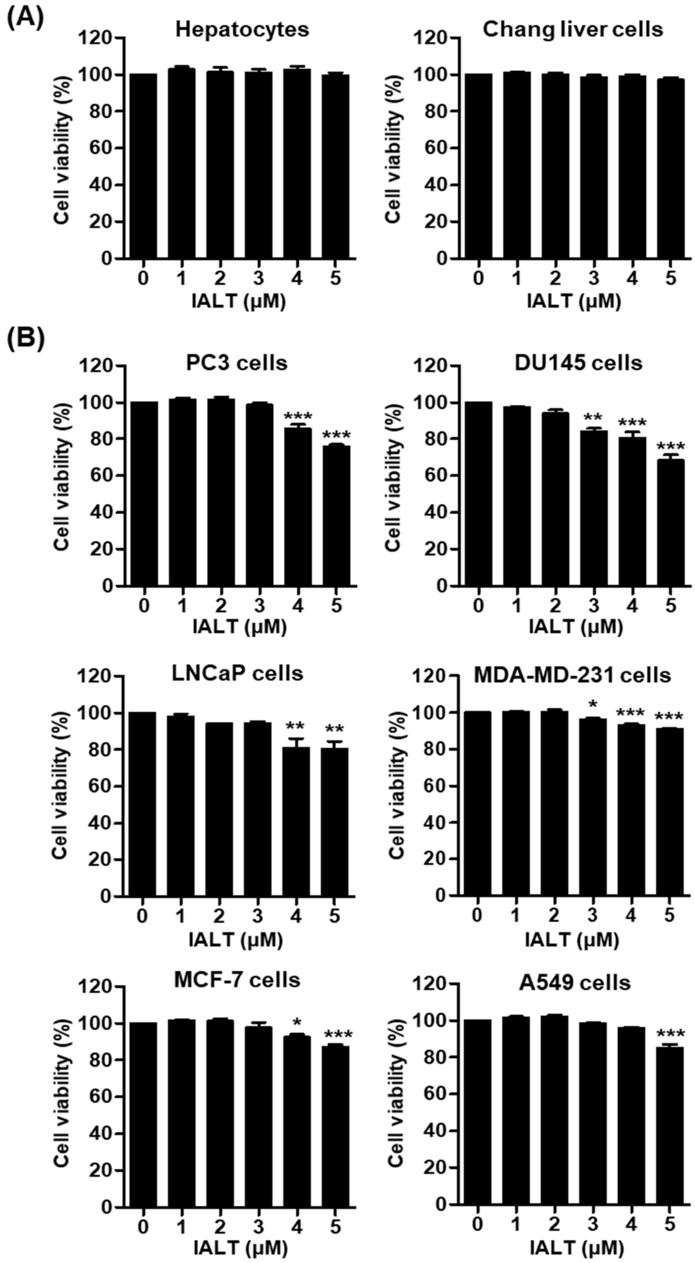

Figure S1. The effect of IALT on cell viability in non-carcinoma cells and various types of carcinoma cells. (A) Non-carcinoma hepatocytes, including primary mouse hepatocytes and Chang liver cells, were treated with the indicated concentration of IALT for 48 h. (B) Three prostate carcinoma cell

lines, including PC3, DU145, and LNCaP, and two breast cancer cell lines, MDA-MD-231 and MCF-7, and human lung adenocarcinoma A549 cells were treated with IALT. (A and B) Cell viability was measured by MTT assay. The data are expressed as mean ± standard deviation (SD) of three independent experiments. * $p < 0.05$, ** $p < 0.01$ and *** $p < 0.001$ vs. untreated control group.

Materials and Methods

Cell Culture

Primary hepatocytes were separated from C57BL/6 mice (6-week-old) and hepatic portal vein was perfused by ethylene glycol-bis (2-aminoethylether)-N,N,N′,N′-tetraacetic acid (EGTA, 50 mL) buffer (5.4 mM KCl, 0.44 mM KH_2PO_4, 140 mM NaCl, 0.34 mM Na_2HPO_4, 0.5 mM EGTA, and 25 mM Tricine) for 10 min, and liver tissue was dispersed using 0.075% collagenase. The inferior vena cava was cut to drain the injected buffer and blood. During perfusion, the inferior vena cava was pressed 2–3 times for 30 s using curved forceps to maintain sufficient flow pressure. Then, the liver tissue was filtered using a 40 μm cell strainer and centrifugated for 5 min at 750 rpm. Hepatocytes were isolated by a 45% Percoll cushion and incubated with Williams E medium (not containing phenol red) containing 10% FBS, 10% horse serum, and 1% antibiotics at 37°C in 5% CO_2. Chang liver cells, human prostate cancer cell lines (DU-145 and PC3), prostate lymph node carcinoma LNCaP cells, human breast carcinoma cell lines (MDA-MB-231 and MCF-7), and human lung adenocarcinoma A549 cells were purchased from the American Type Culture Collection (Manassas, VA, USA). Chang liver cells and DU145 cells were maintained at 37 ℃ in Dulbecco’s Modified Eagle’s Medium (DMEM) supplemented with 10% heat-inactivated fetal bovine serum (FBS), 1% penicillin/streptomycin include 2 mM L-glutamine, 100 U/mL penicillin, and 100 μg/mL streptomycin (WelGENE Inc., Daegu, Republic of Korea) in a humidified atmosphere with 5% CO_2. PC3 cells, LNCaP cells, MDA-MB-231 cells, MCF-7, and A549 cells were grown in RPMI 1640 medium (WelGENE Inc.) supplemented with 10% heat-inactivated FBS (WelGENE Inc.) at 37 ℃ in 5% CO_2 humidified incubator.

Cell Viability Assay

To measure cell viability, the MTT assay was performed, as described previously [46]. Briefly, the cells were incubated with different concentrations of IATL for 48 h. The treated cells were incubated in a medium containing 5 μg/mL MTT solution for 4 h. The medium was carefully discarded, and DMSO was added to each well and gently shaken for 10 min at room temperature (RT). The dissolved formazan was transferred to 96-well plates, and the absorbance was measured at 540 nm by an ELISA microplate reader (Beckman Coulter Inc., Brea, CA, USA).
